# Synthesis and Biological Activity of 3-[Phenyl(1,3-thiazol-2-yl)-amino]propanoic Acids and Their Derivatives

**DOI:** 10.3390/molecules181215000

**Published:** 2013-12-05

**Authors:** Vytautas Mickevičius, Aušra Voskienė, Ilona Jonuškienė, Ramūnė Kolosej, Jūratė Šiugždaitė, Petras Rimantas Venskutonis, Rita Kazernavičiūtė, Zita Brazienė, Elena Jakienė

**Affiliations:** 1 Department of Organic Chemistry, Kaunas University of Technology, Radvilėnų pl. 19, LT-50254 Kaunas, Lithuania; E-Mails: ausravoskiene@yahoo.com (A.V.); ilona.jonuskiene@ktu.lt (I.J.); ramune2004@gmail.com (R.K.); 2 Lithuanian University of Health Sciences, Veterinary Academy, Tilžės 18, LT-47181 Kaunas, Lithuania; E-Mail: jurate.siugzdaite@lva.lt; 3 Department of Food Technology, Kaunas University of Technology, Radvilėnų pl. 19, LT-50254 Kaunas, Lithuania; E-Mails: rimas.venskutonis@ktu.lt (P.R.V.); rita.kazernaviciute@ktu.lt (R.K.); 4 Lithuanian Research Centre for Agriculture and Forestry, Rumokai Experimental Station, Klausučiai, 70462 Vilkaviškis Distr., Lithuania; E-Mail: zitamo421@gmail.com; 5 Institute of Agricultural and Food Sciences, Aleksandras Stulginskis University, Studentų g. 11, LT-53361 Akademija, Kaunor., Lithuania; E-Mail: elena.jakiene@asu.lt

**Keywords:** *N*-phenyl-*N*-thiocarbamoyl-β-alanine, Hantzsch synthesis, thiazole, hydrazone, biological activity

## Abstract

New *N*,*N*-disubstituted β-amino acids and their derivatives with thiazole, aromatic, and heterocyclic substituents were synthesized from *N*-phenyl-*N*-thiocarbamoyl-β-alanine by the Hantzsch method; derivatives with hydrazone fragments were also obtained. Some of the synthesized compounds exhibited discrete antimicrobial activity, and 3-[(4-oxo-4,5-dihydro-1,3-thiazol-2-yl)(phenyl)amino]propanoic acid was found to promote rapeseed growth and to increase seed yield and oil content.

## 1. Introduction

Recently, special attention has been paid to β-amino acids as potential starting materials for bio-organic, medicinal, and natural product chemistry [1]. Nitrogen-containing five- and six-membered heterocyclic compounds and their derivatives, which can be easily synthesized in laboratories are particularly important and often found in natural sources. Synthetic thiazole derivatives possess various biological activities such as anti-inflammatory [2,3], antipyretic [4,5], antiviral [6], anti-microbial [7,8,9,10,11], antifungal [12], and anticancer properties [13,14,15,16]. They have also been used as anticoagulants and antiarrhythmics [16], antidepressants [17], in the treatment of Alzheimer's disease, hypertension [18], schizophrenia [19], hepatitis C [20], allergies [21,22], HIV [23,24], diabetes [25,26], and in functional dye synthesis [27,28]. The 2-aminothiazole structure provides herbicidal [6,29] and antioxidant activities [30]. One of the most convenient methods of thiazole synthesis is the Hantzsch synthesis, which is based on condensation of α-haloketones or aldehydes with thioamides or thiourea. In our previous studies, *N*-aryl-*N*-thiocarbamoyl-β-alanines were used only for the synthesis of 1-substituted 2-thioxotetrahydro-4(1*H*)-pyrimidinones [31,32,33]. The aim of the present study was to synthesize new potentially biologically active *N*,*N*-disubstituted β-amino acids and their derivatives with thiazole, aromatic, heterocyclic fragments and to evaluate their chemical and biological properties.

## 2. Results and Discussion

### 2.1. Synthesis and Structural Peculiarities of New Compounds

3-[(4-Oxo-4,5-dihydro-1,3-thiazol-2-yl)(phenyl)amino]propanoic acid (**2**) was obtained from *N*-phenyl-*N*-thiocarbamoyl-β-alanine (**1**) and monochloroacetic acid in different solvents (water, acetic acid, DMF, ethanol), and by using different bases such as sodium acetate, sodium carbonate, and triethylamine. Reactions in acetic acid and DMF were carried out at 90–100 °C and in ethanol and water, at the mixture boiling temperature for 5 h.

**Scheme 1:**
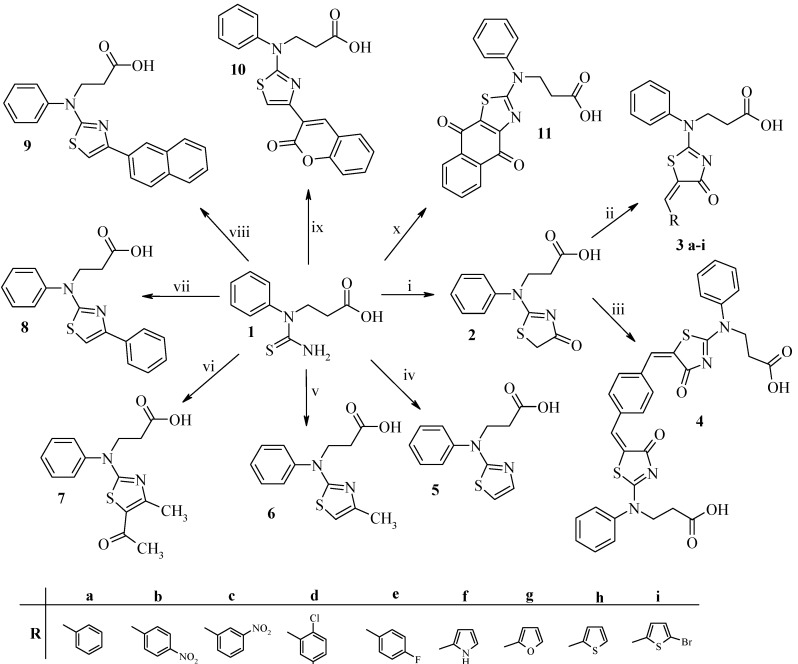
Synthesis of *N*-carboxyethylaminothiazoles and thiazolones **2**–**11**.

The studies showed that the best yield was obtained when reactions were carried out in water in the presence of sodium carbonate. Then the reaction mixture was acidified with acetic acid to pH 6. In order to obtain more thiazole derivatives and to assess the influence of substituents on the biological activity, a series of 3-{[5-substituted 4-oxo-4,5-dihydro-1,3-thiazol-2-yl](phenyl)amino}propanoic acids **3** with aromatic and heterocyclic fragments was synthesized (Scheme 1) . The syntheses were performed by condensation of dihydrothiazolone **2** and aromatic or heterocyclic aldehydes. Since the starting compound **2** and the resulting products **3** are soluble in an alkaline medium, the reactions were carried out in aqueous sodium carbonate solution in the presence of glycine as a bifunctional catalyst. The target products from the reaction mixtures were separated by acidification of the aqueous solutions with acetic acid to pH 6. The preliminary laboratory tests showed that the compounds **2** and **3** had growth regulating effects for rapeseed (*Brassica napus* L.) and St. John’s wort (*Hypericum perforatum* L.) [34]. In the same way, the compound **4** was synthesized from dihydrothiazolone **2** and terephthaldehyde.

*Reagents and Conditions*: (i) ClCH_2_COOH, Na_2_CO_3_, H_2_O, reflux, 5 h, CH_3_COOH; (ii) RCHO, NH_2_CH_2_COOH, Na_2_CO_3_, H_2_O, reflux, 5 h, CH_3_COOH; (iii) terephthalaldehyde, NH_2_CH_2_COOH, Na_2_CO_3_, H_2_O, 5 h, CH_3_COOH; (iv) ClCH_2_CHO, H_2_O, reflux, 2 h, CH_3_COONa; (v) ClCH_2_COCH_3_, CO(CH_3_)_2_, reflux, 1 h, CH_3_COONa; (vi) 3-chloro-2,4-pentanedione, CO(CH_3_)_2_, CH_3_COONa, reflux, 2 h; (vii) 2-bromoacetophenone, CH_3_COONa, 2-propanol, reflux, 5 h; (viii) 2-bromo-2'-acetonaphthone, CH_3_COONa, 2-propanol, reflux, 4 h; (ix) 3-(bromoacetyl)coumarin, CH_3_COONa, 2-propanol, reflux, 7 h; (x) 2,3-dichloro-1,4-naphthoquinone, CH_3_COOH, CH_3_COONa, 80 °C, 24 h.

The structure of the synthesized compounds **3a**–**i** was confirmed by elemental analysis, ^1^H-NMR, ^13^C-NMR and IR spectral data. For example, in the ^1^H-NMR spectrum of compound **3a**, the CH_2_CO and NCH_2_ group proton signals appear at 2.57 and 4.26 ppm, whereas aromatic and CH proton signals are observed in the range of 7.32 to 7.63 ppm. In the ^13^C-NMR spectrum of compound **3a**, the signals of the CH_2_CO and NCH_2_ groups are observed at 34.57 and 51.44 ppm, respectively, whereas aromatic and CH= group carbon signals are observed in the range 111.96–140.42 ppm. The combination of CS, CN, CO, and COOH carbon signals are observed at 133.34, 171.94, 175.79, and 179.19 ppm respectively. In the IR spectrum, the C=N absorption band is observed at 1,540 cm^−^^1^, two C=O bands at 1,686 and 1,725 cm^−^^1^, and a band belonging to the OH group at 3,435 cm^−^^1^ are observed. The formation of isomeric structures in these reactions was not observed. According to data published in the literature, glycine used as a bifunctional catalyst gives a single isomer (*Z*) [35].

3-[Phenyl(1,3-thiazol-2-yl)amino]propanoic acid (**5**) was synthesized from compound **1** and chloroacetaldehyde in refluxing water solution after 2 hours. A water-soluble amino acid hydrochloride is formed, which is present in the base transferred by the addition of sodium acetate to the reaction mixture. The structure of compound **5** is confirmed by its NMR spectral data: in the ^1^H-NMR spectrum two triplets at 2.62 and 4.11 ppm are assigned to the CH_2_CO and NCH_2_ group protons, while two doublets at 6.71 and 7.18 ppm belong to the thiazole ring and the SCH NCH group protons. Aromatic ring proton signals are observed at 7.31–7.50 ppm, and broad proton singlets of the carboxyl group are detectable at 12.30 ppm.

Reactions of **1** with various haloketones were also performed, and a number of new thiazole derivatives **6**–**11** with aliphatic, aromatic, or heterocyclic substituents were thus synthesized. The compounds **6**–**10** were synthesized in acetone or 2-propanol at boiling temperature. Cyclic diketones—quinones—are interesting both for their chemical properties and practical uses. Besides, many compounds containing the quinone group are found in Nature. Various assays showed that these compounds are characterized by a broad spectrum of biological activities [36]. A quinone fragment- containing compound **11** in this work was synthesized from *N*-phenyl-*N*-thiocarbamoyl-β-alanine (**1**) and 2,3-dichloro-1,4-naphthoquinone. The reaction was carried out in acetic acid at 80 °C for 24 h in the presence of sodium acetate. Then the mixture was cooled and diluted with water. The synthesized 3-(*N*-(4,9-dihydro-4,9-dioxonaphtho[2,3-d]thiazol-2-yl)-*N*-phenylamino)propanoic acid (**11**) was purified by dissolving it in aqueous sodium hydroxide solution, filtering, and then acidifying the filtrate with acetic acid to pH 6.

**Scheme 2:**
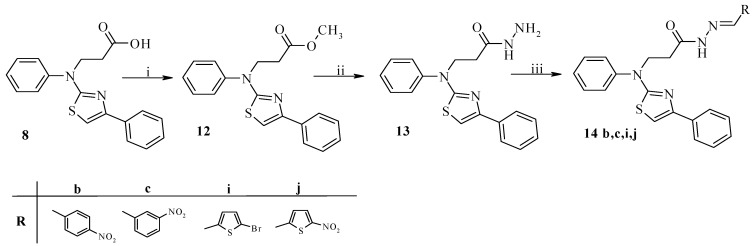
Synthesis of 3-[phenyl(4-phenyl-1,3-thiazol-2-yl)amino]propanoic acid derivatives **12**–**14**.

The compound **8** was modified by introducing a hydrazone fragment into its molecule with the porpose of increasing its antimicrobial properties (Scheme 2). Firstly, acid **8** was esterified with methanol to obtain the corresponding ester **12**, which afterwards was boiled in 2-propanol with hydrazine monohydrate. The resulting acid hydrazide **13** was condensed with aromatic and heterocyclic aldehydes. The NMR spectra of the synthesized hydrazones **14** showed that in DMSO-*d*_6_ solution they exist as a mixture of *E/Z* isomers due the presence of a CO-NH fragment in the molecule and its hindered rotation around the CO-NH bond; however, the *Z* isomer was dominant.

*Reagents and Conditions*: (i) CH_3_OH, H_2_SO_4_, reflux, 6 h; (ii) NH_2_NH_2_, 2-propanol, reflux, 7 h; (iii) RCHO, 2-propanol, reflux, 5 h.

### 2.2. Antimicrobial Evaluation of Synthesized Compounds

The antibacterial activity of the compounds was determined against Gram-positive spore-forming rods of *Bacillus cereus* (ATCC 11778), Gram-positive cocci of *Staphylococcus* (ATCC 9144), Gram-negative rods of *E. coli* (ATCC 8739) and *Pseudomonas aeruginosa* (NCTC 6750) by the broth and spread-plate methods. A range of concentrations for each compound was prepared according to the experimental procedure mentioned in experimental section. The minimal inhibition concentration (MIC, µg/mL) values are listed in Table 1.

**Table 1:** Antibacterial activity *in vitro* of the synthesized compounds (MIC, µg/mL).

Compound	*Staphylococcus aureus*	*Bacillus cereus*	*Esherichia coli*	*Pseudomonas aeruginosa*
2	ni	ni	ni	ni
3a	250	250	250	250
3b	250	250	250	250
3c	250	250	250	250
3d	250	250	250	250
3e	250	250	250	250
3f	250	250	250	250
3g	250	250	250	250
3h	ni	ni	ni	ni
3i	250	250	250	250
5	ni	ni	ni	ni
6	250	250	250	250
7	250	250	250	250
8	250	250	250	250
9	1000	500	1000	ni
10	500	500	500	500
11	250	250	250	250
Oxytetracycline	62.5	62.5	62.5	62.5

ni—no inhibition. The minimum inhibitory concentrations were determined by the micro broth dilution method.

The MIC values of the samples were obtained in spread-plate tests, considering compound concentrations demonstrating antibacterial activity. Antibacterial screening data revealed a prominent inhibitory activity of compounds **3a**–**g**, and **3i** against the tested strains of *S*. *aureus*, *B*. *cereus,* *E*. *coli* and *P*. *aeruginosa* (MIC = 250 µg/mL). The MIC value of 350 µg/mL was obtained against all bacteria for compounds **3a**, **3b**, **3c**, **3d**, **3f**, **3e**, **3i**, **6**, **7**, **8**, and **11**, while for compounds **10** and **9**, depending on bacterial species they were 500 or 1000 µg/mL. Compounds **2**, **5** didn’t show antibacterial activity against the test microorganisms. Based on these data, antibacterial activity of the investigated compounds may be arranged in the following order:

*Staphylococcus aureus*:


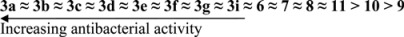


*Bacillus cereus*:


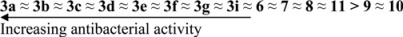


*Esherichia coli*:


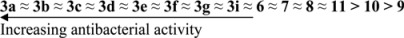


*Pseudomonas aeruginosa*:


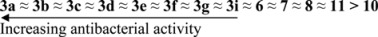


The minimum bactericidal values (MBC) are presented in Table 2. These data also revealed pronounced inhibitory activity of some synthesized compounds against some bacteria species, particulalry *S*. *aureus* and *B*. *cereus* at 250 µg/mL. For instance, the lowest MBC values (250–500 µg/mL) were found for the compounds **3i**, **3g**, **11**. However, the compounds **2**, **3h**, **5** didn’t show any bactericidal activity against the tested microorganisms.

**Table 2:** Bactericidal activity *in vitro* of the synthesized compounds (MBC, µg/mL)

Compound	*Staphylococcus aureus*	*Bacillus cereus*	*Esherichia coli*	*Pseudomonas aeruginosa*
**2**	ni	ni	ni	ni
**3a**	500	1000	500	500
**3b**	500	ni	500	1000
**3c**	500	1000	500	500
**3d**	500	1000	500	500
**3e**	350	1000	500	500
**3f**	500	ni	500	500
**3g**	350	350	350	350
**3h**	ni	ni	ni	ni
**3i**	250	500	500	500
**4**	ni	500	1000	1000
**5**	ni	ni	ni	ni
**6**	500	ni	500	500
**7**	500	ni	500	500
**8**	250	ni	500	500
**9**	1000	1000	1000	ni
**10**	1000	1000	1000	1000
**11**	250	250	250	250
**14 b**	ni	1000	1000	1000
**14 c**	ni	1000	1000	1000
**14 i**	ni	1000	1000	1000
**14 j**	ni	1000	1000	1000
Oxytetracycline	62.5	62.5	250	250

ni—no inhibition; Minimum bactericidal concentrations were determined by using the spread plate method.

Based on these experiments, bactericidal activity of the investigated compounds may be arranged in the following order:

*Decreasing MBC Values against Staphylococcus aureus:*


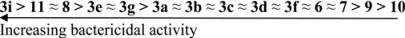


*Decreasing MBC Values against Bacillus cereus*:


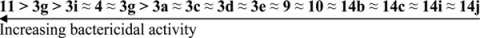


*Decreasing MBC Values against Esherichia coli*:


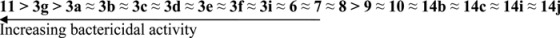


*Decreasing MBC Values against Pseudomonas aeruginosa*:


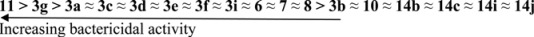


Among the all synthesized compounds, the highest antibacterial activity was exhibited by thiazole compound **11** containing a naphthoquinone ring. Among compounds **3a**–**i**, the highest antibacterial activity was shown by the compounds with furan and bromothiophene substituents in their structure. Aromatic substituents with different groups (NO_2_, F, Cl) didn’t show any inhibiting effect. Among thiazole compounds **5**–**9**, the highest antibacterial activity was exhibited by compound **8** with a phenyl substituent in the 4-position of the thiazole ring.

Attempting to increase antimicrobial activity the structure of compound **8** was modified by replacing the carboxy group by hydrazone and hydrazide derivatives with aromatic and heterocyclic substituents, however, the results obtained suggest that modification of the carboxy group decreases the antimicrobial activity.

It may thus be concluded that the antimicrobial activity of the tested compounds depends on their structural peculiarities. 3-(*N*-(4,9-dihydro-4,9-dioxonaphtho[2,3-d]thiazol-2-yl)-*N*-phenylamino)propanoic acid (**11**), 3-(*N*-(5-((furan-2-yl)methylene)-4,5-dihydro-4-oxothiazol-2-yl)-*N*-phenylamino)propanoic acid (**3g**), and 3-(*N*-(5-((5-bromothiophen-2-yl)methylene)-4,5-dihydro-4-oxothiazol-2-yl)-*N*-phenyl- amino)propanoic acid (**3i**) were found to exhibit a promising antibacterial activity.

### 2.3. Evaluation of Synthesized Compounds on Rapeseed Yield and Biochemical Content

The effect of 3-[(4-oxo-4,5-dihydro-1,3-thiazol-2-yl)(phenyl)amino]propanoic acid (**2**) on the rapeseed yield and biochemical content is presented in Table 3. When rapeseed seedlings were sprayed with solutions of **2** of various concentrations, the yield of seeds ranged from 2.07 to 2.44 t/ha, *i.e.*, 19%–40% higher in comparison with the control sample. The highest seed yield was obtained when rapeseed seedlings were sprayed with 150 mg/L of the test compound solution. Other quality characteristics were also affected by spraying rapeseed seedlings with the test compound; for instance, the highest mass of 1,000 seeds (4.17 g) was determined when 25 mg/L were applied, whereas at the same concentration the oil content increased by 13.3%–39.3% in comparison with the control sample.

**Table 3:** The effect of 3-[(4-oxo-4,5-dihydro-1,3-thiazol-2-yl)(phenyl)amino]propanoic acid (**2**) on rapeseed yield and biochemical content.

	The concentration of 3-[(4-oxo-4,5-dihydro-1,3-thiazol-2-yl)(phenyl)amino]propanoic acid (2), mg/L						
	0	25	50	75	100	125	150
Yield, t/ha	1.74 ± 0.11	2.07 ± 0.23	2.21 ± 0.08	2.11 ± 0.28	2.20 ± 0.08	2.41 ± 0.11	2.44 ± 0.20
Seed mass (per 1,000 seeds), g	3.94 ± 0.16	4.17 ± 0.10	4.02 ± 0.12	3.81 ± 0.16	3.96 ± 0.09	3.85 ± 0.14	3.94 ± 0.21
Oil content, kg/t	239.9 ± 1.7	283.4 ± 8.3	271.8 ± 7.5	334.3 ± 5.7	301.2 ± 4.9	281.8 ± 7.9	296.7 ± 6.5
Protein content, mg/100g	15.9 ± 0.1	27.1 ± 0.3	30.7 ± 0.3	31.2 ± 0.3	30.3 ± 0.3	22.8 ± 0.4	22.5 ± 0.2
Ash, %	4.53 ± 0.02	4.53 ± 0.05	4.48 ± 0.04	4.23 ± 0.10	4.16 ± 0.10	4.07 ± 0.14	4.10 ± 0.13

Application of 3-[(4-oxo-4,5-dihydro-1,3-thiazol-2-yl)(phenyl)amino]propanoic acid (**2**) increased both the content of oil and protein. The highest content of oil (334 kg/t) and protein (31.2 mg/100g) was obtained upon using the 75 mg/L concentration of the study compound solution. The content of protein varied from 15.9 to 31.2 mg/100 g. When rapeseed seedlings were sprayed with 75 mg/L of the test compound solution, protein content was two times higher in comparison with the control sample.

The effect of 3-[(4-oxo-4,5-dihydro-1,3-thiazol-2-yl)(phenyl)amino]propanoic acid (**2**) on the composition of fatty acids in rapeseed is presented in Table 4. The results showed that the variation of oleic acid content was insignificant (−0.09 to +0.77%) in comparison with the control sample. When rapeseed had been sprayed with 50 mg/L of the aforementioned solution, the highest content of oleic acid (61.51%) was obtained in the seeds. The content of saturated fatty acids (palmitic and stearic) varied insignificantly in comparison with the control sample. When rapeseed had been sprayed with the test compound solutions at various concentrations, some increase in the content of linolenic acid was observed; however, it was insignificant. Upon spraying rapeseed seedlings with 75 mg/L of the study compound solution, the highest content of linoleic acid (20.96%) was obtained in the seeds.

**Table 4:** The effect of 3-[(4-oxo-4,5-dihydro-1,3-thiazol-2-yl)(phenyl)amino]propanoic acid (**2**) on the content of fatty acids in rapeseed.

Fatty acid, %	The concentration of 3-[(4-oxo-4,5-dihydro-1,3-thiazol-2-yl)(phenyl)amino]propanoic acid (2), mg/L						
	0	25	50	75	100	125	150
Palmitic	5.11 ± 0.05	4.57 ± 0.06	4.79 ± 0.22	4.59 ± 0.07	5.23 ± 0.31	4.90 ± 0.09	4.76 ± 0.03
Stearic	2.11 ± 0.08	2.02 ± 0.02	2.07 ± 0.01	2.02 ± 0.01	2.05 ± 0.01	2.04 ± 0.01	2.03 ± 0.01
Oleic	61.04 ± 0.26	61.07 ± 0.09	61.51 ± 0.03	61.28 ± 0.02	60.98 ± 0.08	61.21 ± 0.18	61.41 ± 0.00
Linoleic	20.55 ± 0.13	20.83 ± 0.09	20.61 ± 0.01	20.96 ± 0.06	20.93 ± 0.02	20.84 ± 0.05	20.85 ± 0.5
Eicosenoic	1.02 ± 0.08	1.09 ± 0.03	1.07 ± 0.04	1.05 ± 0.01	0.93 ± 0.07	1.02 ± 0.02	0.99 ± 0.01
Linolenic	8.49 ± 0.03	8.75 ± 0.02	8.54 ± 0.02	8.66 ± 0.02	8.67 ± 0.01	8.64 ± 0.01	8.60 ± 0.02

Summarizing the data of the field experiments spraying rapeseed seedlings with different concentrations of 3-[(4-oxo-4,5-dihydro-1,3-thiazol-2-yl)(phenyl)amino]propanoic acid (**2**) solutions resulted in the following:

(a) 100 mg/L: the highest number of pods and siliques was obtained;
(b) 150 mg/L: the highest seed yield was obtained;
(c) 25 mg/L: the highest seed mass was obtained.

**Scheme 3:**
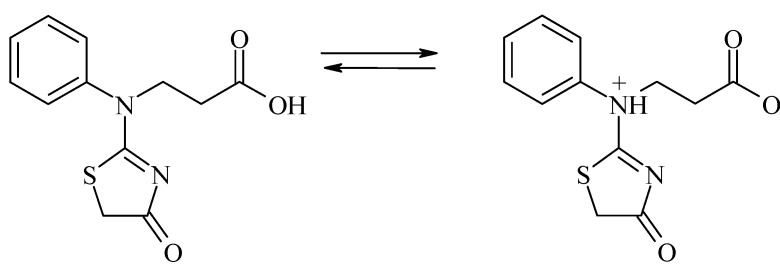
The possible forms of compound **2.**

During osmotic stress plants accumulate in the cytoplasm of their cells organic osmolytes such as proline, valine, isoleucine, ectoine, aspartic acid, betaine, glucose, fructose, sucrose, fructans, mannitol, pinitol, and myo-inositol (inositol) [37]. The regulating effect of compound **2** on rapeseed could be explained by the properties of typical structure osmolytes: they could exist in the zwitterionic form (Scheme 3).

## 3. Experimental

### 3.1. General

The starting materials and solvents were obtained from Sigma-Aldrich Chemie GmbH (Munich, Germany) and Fluka (Buchs, Switzerland) and were used without further purification. The methods used to follow the reactions were TLC and NMR. The NMR spectra were recorded on a Varian Unity Inova (300 MHz) spectrometer (Varian, Inc., Palo Alto, CA, USA) and Bruker Avance III/400 (400 MHz). Chemical shifts are expressed as δ, ppm relative to TMS. The *J* constants are given in Hz. The IR spectra (ν, cm^−1^) were recorded on a Perkin–Elmer BX FT–IR spectrometer (Perkin–Elmer Inc., Waltham, MA, USA) using KBr tablets. Mass spectra were obtained on a Waters ZQ 2000 spectrometer (Waters, Eschborn, Germany) using the electrospray ionization (ESI) mode and operating at 25 V. Elemental analyses were performed with a CE-440 elemental analyzer (Exeter Analytical Inc., Chelmsford, MA, USA). Melting points were determined with a B-540 Melting Point Analyzer (Büchi Corporation, New Castle, DE, USA) and are uncorrected. TLC was performed using Merck, Silica gel 60 F254 (Kieselgel 60 F254) plates.

*3-[(4-Oxo-4,5-dihydro-1,3-thiazol-2-yl)(phenyl)amino]propanoic Acid* (**2**). A mixture of thioureido acid **1** (2.24 g, 0.01 mol), chloroacetic acid (1.04 g, 0.011 mol), sodium carbonate (1.16 g, 0.011 mol) and water (30 mL) was refluxed for 5 h. Then the mixture was acidified with acetic acid (6 mL) to pH 6. The precipitate was filtered off, washed with water, and crystallized. Yield 1.78 g, 68%; light yellow crystals m.p. 195–196 °C (from 2-propanol) (198–199 °C) [38]; IR (KBr): *ν* (cm^−1^) 3426 (OH), 1723, 1659 (CO); ^1^H-NMR (DMSO-*d*_6_): δ 2.58 (t, *J* = 7.4 Hz, 2H, CH_2_CO), 3.92 (s, 2H, SCH_2_), 4.17 (t, *J* = 7.3 Hz, 2H, NCH_2_), 7.41–7.57 (m, 5H, H_Ar_), 12.42 (s, 1H, OH); ^13^C-NMR (DMSO-d_6_): δ 31.9 (CH_2_CO), 40.5 (SCH_2_), 49.9 (NCH_2_), 128.0, 129.6, 129.8, 140.1 (C_Ar_), 171.9, 183.1 (CO), 186.9 (C=N); Anal. Calcd for C_12_H_12_N_2_O_3_S: C, 54.53%; H, 4.58%; N, 10.60%; Found: C, 54.69%; H, 4.56%; N, 10.68%.

### 3.2. General Procedure for Preparation of 3-{[(5Z)-5-Substituted 4-oxo-4,5-dihydro-1,3-thiazol-2-yl](phenyl)amino}propanoic Acids **3a**–**i**

A mixture of compound **2** (0.52 g, 2 mmol), the corresponding aldehyde (2.2 mmol), glycine (0.15 g, 2 mmol), and sodium carbonate (0.21 g, 2 mmol), water (6 mL) was refluxed for 5 h. Then the reaction mixture was diluted with water (50 mL) and acidified with acetic acid to pH 6. The precipitate was filtered and washed with water. Purification was performed by dissolving the crystals in 10% aqueous NaOH (100 mL), filtering, and acidifying the filtrate with acetic acid to pH 6 (the procedure was repeated 2 times).

*3-{[(5Z)-5-Benzylidene-4-oxo-4,5-dihydro-1,3-thiazol-2-yl](phenyl)amino}propanoic Acid* (**3a**). Yellow crystals, (0.52 g, 76%), m.p. 175–176 °C; IR (KBr): *ν* (cm^−1^) 3435 (OH), 1725, 1686 (CO), 1540 (CN); ^1^H-NMR (DMSO-*d*_6_): δ 2.57 (t, *J* = 7.4 Hz, 2H, CH_2_CO), 4.26 (t, *J* = 7.5 Hz, 2H, NCH_2_), 7.32–7.63 (m, 11H, H_Ar_+CH); ^13^C-NMR (DMSO-*d*_6_): δ 32.1 (CH_2_CO), 50.1 (NCH_2_), 127.9, 128.9, 129.1, 129.5, 129.7, 130.1, 133.3, 139.6 (C_Ar_, C_thiazole_, CH), 172.0, 175.8 (CO), 179.2 (C=N); Anal. Calcd for C_19_H_16_N_2_O_3_S: C, 64.76%; H, 4.58%; N, 7.95%; Found: C, 64.44%; H, 4.63%; N, 8.04%.

*3-{[(5Z)-5-(4-Nitrobenzylidene)-4-oxo-4,5-dihydro-1,3-thiazol-2-yl](phenyl)amino}propanoic Acid* (**3b**). Yellow crystals, (0.57 g, 73%), m.p. 208–209 °C; IR (KBr): *ν* (cm^−1^) 3058 (OH), 1729, 1699 (CO), 1539 (CN); ^1^H-NMR (DMSO-*d*_6_): δ 2.34 (t, *J* = 7.8 Hz, 2H, CH_2_CO), 4.20 (t, *J =* 7.9 Hz, 2H, NCH_2_), 7.32–7.64 (m, 10 H, H_Ar_+CH); ^13^C-NMR (DMSO-*d*_6_): δ 34.5 (CH_2_CO), 52.0 (NCH_2_), 115.7, 124.2, 127.8, 128.1, 130.3, 133.3, 139.6, 140.4, 147.0 (C_Ar_, C_thiazole_, CH), 172.2, 175.9 (CO), 179.0 (C=N); Anal. Calcd for C_19_H_15_N_3_O_5_S: C, 57.42%; H, 3.80%; N, 10.57%; Found: C, 56.94%; H, 3.75%; N, 10.86%.

*3-{[(5Z)-5-(3-Nitrobenzylidene)-4-oxo-4,5-dihydro-1,3-thiazol-2-yl](phenyl)amino}propanoic Acid* (**3c**). Yellow crystals, (0.50 g, 64%), m.p. 150–151 °C; IR (KBr): *ν* (cm^−1^) 3074 (OH), 1731, 1709 (CO), 1531 (CN); ^1^H-NMR (DMSO-*d*_6_): δ 2.59 (t, *J* = 7.5 Hz, 2H, CH_2_CO), 4.28 (t, *J* = 7.5 Hz, 2H, NCH_2_), 7.55–8.43 (m, 10H, H_Ar_+CH); ^13^C-NMR (DMSO-d_6_): δ 32.7 (CH_2_CO), 50.9 (NCH_2_), 124.4, 128.5, 128.6, 130.6, 131.3, 132.4, 135.4, 135.9, 140.2, 148.6 (C_Ar_, C_thiazole_, CH), 172.6, 176.2 (CO), 179.6 (C=N); Anal. Calcd for C_19_H_15_N_3_O_5_S: C, 57.42%; H, 3.80%; N, 10.57%; Found: C, 57.39%; H, 3.77%; N, 10.51%.

*3-{[(5Z)-5-(2-Chloro-4-nitrobenzylidene)-4-oxo-4,5-dihydro-1,3-thiazol-2-yl](phenyl)amino}propanoic Acid* (**3d**). Yellow crystals, (0.53 g, 62%), m.p. 155–156 °C; IR (KBr): *ν* (cm^−1^) 3071 (OH), 1735, 1698 (CO), 1529 (CN); ^1^H-NMR (DMSO-*d*_6_): δ 2.63 (t, *J* = 7.4 Hz, 2H, CH_2_CO), 4.30 (t, *J* = 7.4 Hz, 2H, NCH_2_), 7.53–8.22 (m, 9H, H_Ar_+CH); ^13^C-NMR (DMSO-d_6_): δ 31.9 (CH_2_CO), 50.4 (NCH_2_), 122.9, 123.6, 125.4, 128.2, 130.2, 130.3, 131.6, 133.4, 135.3, 139.6, 140.3, 146.6 (C_Ar_, C_thiazole_, CH), 172.0, 175.9 (CO), 178.6 (C=N); Anal. Calcd for C_19_H_14_ClN_3_O_5_S: C, 52.84%; H, 3.27%; N, 9.73%; Found: C, 52.78%; H, 3.33%; N, 9.78%.

*3-{[(5Z)-5-(4-Fluorobenzylidene)-4-oxo-4,5-dihydro-1,3-thiazol-2-yl](phenyl)amino}propanoic Acid* (**3e**). Yellow crystals, (0.62 g, 85%), m.p. 167–168 °C; IR (KBr): *ν* (cm^−1^) 3050 (OH), 1736, 1701 (CO), 1508 (CN); ^1^H-NMR (DMSO-*d*_6_): δ 2.61 (t, *J* = 7.2 Hz, 2H, CH_2_CO), 4.10 (t, *J* = 7.5 Hz, 2H, NCH_2_), 6.71–7.52 (m, 10H, H_Ar_+CH), 12.29 (OH); ^13^C-NMR (DMSO-d_6_): δ 32.7 (CH_2_CO), 50.5 (NCH_2_), 116.1, 116.4, 128.1, 128.8, 129.1, 129.9, 130.0, 131.7, 139.9, 160.7 (C_Ar_, C_thiazole_, CH) 172.47, 175.8 (CO), 179.4 (C=N); Anal. Calcd for C_19_H_15_FN_2_O_3_S: C, 61.61%; H, 4.08%; N, 7.56%; Found: C, 61.77%; H, 3.91%; N, 7.48%.

*3-{[(5Z)-4-oxo-5-(1H-Pyrrol-2-ylmethylene)-4,5-dihydro-1,3-thiazol-2-yl](phenyl)amino}propanoic* *Acid* (**3f**). Yellow crystals, (0.46 g, 69%), m.p. 248–249 °C; IR (KBr): *ν* (cm^−1^) 3064 (OH), 1715, 1673 (CO), 1530 (CN); ^1^H-NMR (DMSO-*d*_6_): δ 2.28 (t, *J* = 8.3 Hz, 2H, CH_2_CO), 4.15 (t, *J* = 8.2 Hz, 2H, NCH_2_), 6.03–7.66 (m, 9H, H_Ar_, CH, CH_pyrrole_), 11.79 (NH); ^13^C-NMR (DMSO-d_6_): δ 35.4 (CH_2_CO), 52.0 (NCH_2_), 111.2, 111.9, 120.5, 122.1, 123.3, 127.6, 128.3, 129.6, 130.0, 140.6 (C_Ar_, C_thiazole_, CH, C_pyrrole_) 174.3, 174.7 (CO), 180.1 (C=N); Anal. Calcd for C_17_H_15_N_3_O_3_S: C, 59.81%; H, 4.43%; N, 12.31%; Found: C, 59.75%; H, 4.39%; N, 12.22%.

*3-{[(5Z)-5-(2-Furylmethylene)-4-oxo-4,5-dihydro-1,3-thiazol-2-yl](phenyl)amino}propanoic Acid* (**3g**). Brown crystals, (0.41 g, 61%), m.p. 139–140 °C; IR (KBr): *ν* (cm^−1^) 3050 (OH), 1727, 1682 (CO), 1532 (CN); ^1^H-NMR (DMSO-*d*_6_): δ 2.61 (t, *J* = 7.4 Hz, 2H, CH_2_CO), 4.25 (t, *J* = 7.4 Hz, 2H, NCH_2_), 6.61–7.91 (m, 9H, H_Ar_, CH, H_furan_); ^13^C-NMR (DMSO-*d*_6_): δ 32.0 (CH_2_CO), 49.8 (NCH_2_), 113.3, 117.0, 117.2, 126.4, 128.3, 130.0, 130.1, 140.0, 146.6, 149.5 (C_Ar_, C_thiazole_, CH, C_furan_), 172.1, 176.8 (CO), 179.4 (C=N); Anal. Calcd for C_17_H_14_N_2_O_4_S: C, 59.64%; H, 4.12%; N, 8.18%; Found: C, 59.58% H, 4.21%; N, 8.22%.

*3-{[(5Z)-4-Oxo-5-(2-thienylmethylene)-4,5-dihydro-1,3-thiazol-2-yl](phenyl)amino}propanoic Acid* (**3h**). Yellow crystals (0.48 g, 68%), m.p. 137–138 °C; IR (KBr): *ν* (cm^−1^) 3057 (OH), 1726, 1678 (CO), 1542 (CN); ^1^H-NMR (DMSO-*d*_6_): δ 2.62 (t, *J* = 7.4 Hz, 2H, CH_2_CO), 4.26 (t, *J* = 7.4 Hz, 2H, NCH_2_), 7.16–7.89 (m, 9H, H_Ar_+CH, H_thiophene_); ^13^C-NMR (DMSO-*d*_6_): δ 32.0 (CH_2_CO), 50.0 (NCH_2_), 123.5, 127.2, 128.3, 128.9, 130.0, 130.1, 131.5, 133.5, 138.4, 140.0 (C_Ar_, C_thiazole_, CH, C_thiophene_), 172.1, 175.3 (CO), 179.3 (C=N); Anal. Calcd for C_17_H_14_N_2_O_3_S_2_: C, 56.96%; H, 3.94%; N, 7.82%; Found: C, 57.02%; H, 3.88%; N, 7.89%.

*3-[{(5Z)-5-[(5-Bromo-2-thienyl)methylene]-4-oxo-4,5-dihydro-1,3-thiazol-2-yl}(phenyl)amino]propanoic Acid* (**3i**). Yellow crystals (0.47 g, 55%), m.p. 143–144 °C; IR (KBr): *ν* (cm^−1^) 3007 (OH), 1723, 1682 (CO), 1538 (CN); ^1^H-NMR (DMSO-*d*_6_): δ 2.57 (t, *J* = 7.5 Hz, 2H, CH_2_CO), 4.25 (t, *J* = 7.5 Hz, 2H, NCH_2_), 7.28–7.84 (m, 8H, H_Ar_, CH, H_thiophene_); ^13^C-NMR (DMSO-*d*_6_): δ 32.4 (CH_2_CO), 50.4 (NCH_2_), 116.9, 122.41, 124.4, 128.0, 128.2, 130.1, 132.3, 133.8, 139.9, 140.2 (C_Ar_, C_thiazole_, CH, C_thiophene_), 172.4, 174.8 (CO), 179.0 (C=N); Anal. Calcd for C_17_H_13_BrN_2_O_3_S_2_: C, 46.69%; H, 3.00%; N, 6.41%; Found: C, 46.77%; H, 2.97%; N, 6.50%.

*3-({5-(4-{[2-[(3-Hydroxy-3-oxopropyl)aniline]* *-4-oxo-1,3-thiazol-5-yliden]methyl}phenyl)methyliden]-4-oxo-4,5-dihydro-1,3-thiazol-2-yl}aniline)propanoic Acid* (**4**). A mixture of compound **2** (0.61 g, 2.3 mmol), terephthalaldehyde (0.15 g, 1.14 mmol), glycine (0.17 g, 2.3 mmol), sodium carbonate (0.49 g, 4.6 mmol) and water (7 mL) was refluxed for 5 h. Then the reaction mixture was diluted with water (50 mL) and acidified with acetic acid to pH 6. The precipitate was filtered and washed with water. Purification was performed by dissolving the crystals in 2% aqueous KOH (100 mL), filtering, and acidifying the filtrate with acetic acid to pH 6. Yellow crystals, (0.34 g, 27%), m.p. 269–270 °C; IR (KBr): ν (cm^−1^) 3023 (OH), 1729, 1710, 1694, 1688 (CO), 1532, 1538 (CN); ^1^H-NMR (DMSO-*d*_6_): δ 2.61 (t, *J* = 7.2 Hz, 4H, CH_2_CO), 4.27 (t, *J* = 7.1 Hz, 4 H, NCH_2_), 7.46–7.65 (m, 16H, C_Ar_, CH); Anal. Calcd for C_32_H_26_N_4_O_6_S_2_: C, 61.33%; H, 4.18%; N, 8.94%; Found: C, 61.30%; H, 4.07%; N, 8.88%.

*3-[Phenyl(1,3-thiazol-2-yl)amino]propanoic Acid* (**5**). A mixture of the thioureido acid **2** (1.12 g, 5 mmol), chloroacetaldehyde 50% aqueous solution (0.79 g, 0.01 mol), water (20 mL) was refluxed for 2 h. Then sodium acetate (0.82 g, 0.01 mol) was added and the mixture was stirred for 5 min. The precipitate was filtered and washed with water. Purification was performed by dissolving the crystals in 10% aqueous sodium carbonate (75 mL), filtering, and acidifying the filtrate with acetic acid to pH 6 (the procedure was repeated 2 times). Yellow crystals, (0.50 g, 42%), m.p. 144–145 °C; IR (KBr): *ν* (cm^−1^) 3076 (OH), 1717 (CO), 1514 (CN); ^1^H-NMR (DMSO-*d*_6_): δ 2.62 (t, *J* = 7.2 Hz, 2H, CH_2_CO), 4.11 (t, *J* = 7.5 Hz, 2H, NCH_2_), 6.71 (d, *J* = 3.6 Hz, 1H, NCH), 7.18 (d, *J* = 3.6 Hz, 1H, SCH), 7.31–7.50 (m, 5H, H_Ar_), 12.30 (br s, 1H, OH); ^13^C-NMR (DMSO-*d*_6_): δ 32.2 (CH_2_CO), 48.5 (NCH_2_), 108.1, 126.6, 127.2, 130.0, 139.1, 144.8 (C_Ar_, C_thiazole_) 169.5 (CO), 172.5 (C=N); Anal. Calcd for C_12_H_12_N_2_O_2_S: C, 58.05%; H, 4.87%; N, 11.28%; Found: C, 58.11%; H, 4.93%; N, 11.31%.

*3-[(4-Methyl-1,3-thiazol-2-yl)(phenyl)amino]propanoic Acid* (**6**). A mixture of thioureido acid **1** (0.56 g, 2.5 mmol), chloroacetone (0.29 g, 3 mmol) in acetone (10 mL) was refluxed for 1 h and diluted with water (50 mL). Then sodium acetate (0.98 g, 12 mmol) was added, and the mixture was stirred for 5 min. The precipitate was filtered, and washed with water. Yellow crystals (0.52 g, 79%), m.p. 149–150 °C (from acetone-water mixture); IR (KBr): *ν* (cm^−1^) 3128 (OH), 1712 (CO), 1515 (CN); ^1^H-NMR (DMSO-*d*_6_): δ 2.16 (s, 3H, CH_3_), 2.60 (t, *J* = 7.3 Hz, 2H, CH_2_CO), 4.09 (t, *J* = 7.4 Hz, 2H, NCH_2_), 6.26 (s, 1H, SCH), 7.30–7.49 (m, 5H, H_Ar_), 12.29 (br s, 1H, OH); ^13^C-NMR (DMSO-*d*_6_): δ 17.4 (CH_3_), 32.3 (CH_2_CO), 48.3 (NCH_2_), 102.1, 126.8, 127.2, 129.9, 144.5, 148.3 (C_Ar_, C_thiazole_); 168.67 (CO), 172.6 (C=N); Anal. Calcd for C_13_H_14_N_2_O_2_S: C, 59.52%; H, 5.38%; N, 10.68%; Found: C, 59.47%; H, 5.45%; N, 10.55%.

*3-[(5-Acetyl-4-methyl-1,3-thiazol-2-yl)(phenyl)amino]propanoic Acid* (**7**). A mixture of thioureido acid **1** (1.0 g, 4.5 mmol), 3-chloro-2,4-pentanedione (0.76 g, 5.6 mmol), acetone (10 mL) was refluxed for 2 h and diluted with water (50 mL). Then sodium acetate ((1.8 g, 22 mmol) was added, and the mixture was stirred for 5 min. The precipitate was filtered and washed with water. Purification was performed by dissolving the crystals in 10% aqueous sodium hydroxide (50 mL), filtering, and acidifying the filtrate with acetic acid to pH 6. Light yellow crystals, (0.84 g, 92%), m.p. 175–176 °C; IR (KBr): *ν* (cm^−1^) 1721, 1700 (CO), 1576 (C=N); ^1^H-NMR (DMSO-*d*_6_): δ 2.31 (s, 3H, CH_3_), 2.48 (s, 3H, CH_3_), 2.51 (t, *J* = 7.2 Hz, 2H, CH_2_CO), 4.15 (t, *J* = 7.3 Hz, 2H, NCH_2_), 7.44–7.53 (m, 6H, H_Ar_, H_thiazole_), 12.36 (s, 1H, OH); Anal. Calcd for C_15_H_16_N_2_O_3_S: C, 59.19%; H, 5.30%; N, 9.20%; Found: C, 59.26%; H, 5.15%; N, 9.11%.

*3-[Phenyl(4-phenyl-1,3-thiazol-2-yl)amino]propanoic Acid* (**8**). A mixture of thioureido acid **2** (1.12 g, 5 mmol), 2-bromoacetophenone (0.99 g, 5 mmol), sodium acetate (0.82 g, 10 mmol), 2-propanol (10 mL) was refluxed for 5 h. The mixture was diluted with water (30 mL). The precipitate was filtered and washed with water. Purification was performed by dissolving the crystals in 10% aqueous KOH (30 mL), filtering, and acidifying the filtrate with acetic acid to pH 6. Yellow crystals, (1.56 g, 96%), m.p. 101–102 °C; IR (KBr): *ν* (cm^−1^) 3416 (OH), 1712 (CO), 1508 (CN); ^1^H-NMR (DMSO-*d*_6_): δ 2.66 (t, *J* = 7.3 Hz, 2H, CH_2_CO), 4.19 (t, *J* = 7.5 Hz, 2H, NCH_2_), 7.13 (s, 1H, SCH), 7.26–7.88 (m, 10H, H_Ar_); ^13^C-NMR (DMSO-*d*_6_): δ 32.8 (CH_2_CO), 48.9 (NCH_2_), 102.9, 125.6, 126.7, 127.3, 127.5, 128.5, 129.9, 134.6, 150.3, 144.6 (C_Ar, _C_thiazole_), 168.7 (CO), 173.0 (C=N); Anal. Calcd for C_18_H_16_N_2_O_2_S: C, 66.64%; H, 4.97%; N, 8.64%; Found: C, 66.60%; H, 4.83%; N, 8.57%.

*3-{[4-(2-Naphthyl)-1,3-thiazol-2-yl]* *(phenyl)amino}propanoic Acid* (**9**). A mixture of thioureido acid **2** (1.12 g, 5 mmol), 2-bromoacetonaphthone (1.25 g, 5 mmol), 2-propanol (10 mL), sodium acetate (0.82 g, 10 mmol) was refluxed for 4 h. The mixture was diluted with water. The precipitate was filtered and washed with water. Purification was performed by dissolving the crystals in 10% aqueous K_2_CO_3_ (50 mL), filtering, and acidifying the filtrate with acetic acid to pH 6. Yellow crystals, (1.52 g, 82%), m.p. 143–144 °C; IR (KBr): *ν* (cm^−1^) 3112 (OH), 1741 (CO), 1511 (CN); ^1^H-NMR (DMSO-*d*_6_): δ 2.67 (t, *J* = 7.4 Hz, 2H, CH_2_CO), 4.25 (t, *J* = 7.4 Hz, 2H, NCH_2_), 7.30 (s, 1H, SCH), 7.48–7.88 (m, 12H, H_Ar_); Anal. Calcd for C_22_H_18_N_2_O_2_S: C, 70.57%; H, 4.85%; N, 7.48%; Found: C, 70.41%; H, 4.92%; N, 7.39%.

*3-{[4-(2-Oxo-2H-chromen-3-yl)-1,3-thiazol-2-yl](phenyl)amino}propanoic Acid* (**10**). A mixture of thioureido acid **2** (1.12 g, 5 mmol), 3-(bromoacetyl)coumarin (1.34 g, 5 mmol), sodium acetate (0.82 g, 10 mmol), 2-propanol (10 mL) was refluxed for 7 h. After cooling, the precipitate was filtered and washed with water. Purification was performed by dissolving the crystals in 10% aqueous KOH (50 mL), filtering, and acidifying the filtrate with acetic acid to pH 6. Light brown crystals (1.68 g, 86%), m.p. 172–173 °C; IR (KBr): *ν* (cm^−1^) 3137 (OH), 1717, 1709 (CO), 1536 (CN); ^1^H-NMR (DMSO-*d*_6_): δ 2.65 (t, *J* = 7.3 Hz, 2H, CH_2_CO), 4.23 (t, *J* = 7.3 Hz, 2H, NCH_2_), 7.34–7.89 (m, 10H, H_Ar_), 8.64 (s, 1H, SCH); Anal. Calcd for C_21_H_18_N_2_O_4_S: C, 63.95%; H, 4.60%; N, 7.10%; Found: C, 64.11%; H, 4.66%; N, 7.08%.

*3-(N-(4,9-Dihydro-4,9-dioxonaphtho[2,3-d]* *thiazol-2-yl)-N-phenylamino)propanoic Acid* (**11**). A mixture of thioureido acid **2** (2.24 g, 0.01 mol), 2,3-dichloro-1,4-naphthoquinone (2.72 g, 12 mmol), sodium acetate (2.95 g, 36 mmol), acetic acid (50 mL) was stirred at 80 °C for 24 h. After cooling mixture was diluted with water (100 mL). The precipitate formed was filtered and washed with water. Purification was performed by dissolving the crystals in 10% aqueous KOH (350 mL), filtering, and acidifying the filtrate with acetic acid to pH 6. Claret crystals (2.38 g, 62%), m.p. 185 °C (*decomp*.); IR (KBr): *ν* (cm^−1^) 3063 (OH), 1712, 1634, 1622 (CO), 1527 (CN); ^1^H-NMR (DMSO-*d*_6_): δ 2.71 (t, *J* = 7.1 Hz, 2H, CH_2_CO), 4.53 (t, *J* = 7.1 Hz, 2H, NCH_2_), 7.43–7.91 (m, 9H, H_Ar_), 12.19 (br s, 1H, OH); ^13^C-NMR (DMSO-*d*_6_): δ 32.1 (CH_2_CO), 49.1 (NCH_2_), 120.6, 125.8, 127.0, 128.7, 129.3, 130.5, 131.7, 135.0, 142.6, 160.1 169.8 (C_Ar_, C_thiazole_), 172.1, 175.3, 179.8 (CO); Anal. Calcd for C_20_H_14_N_2_O_4_S: C, 63.48%; H, 3.73%; N, 7.40%; Found: C, 63.36%; H, 3.62%; N, 7.44%.

*Methyl 3-[phenyl(4-phenyl-1,3-thiazol-2-yl)amino]propanoate* (**12**). A mixture of 3-(*N*-phenyl-*N*-(4-phenylthiazol-2-yl)amino)propanoic acid (**8**) (8.08 g, 0.025 mol), methanol (150 mL), and H_2_SO_4_ (2 mL) was refluxed for 6 h. Then the solvent was evaporated. The precipitated product was neutralized with 5% sodium bicarbonate solution and extracted with diethyl ether (3 times with 100 mL); then the ether was evaporated. Oil, (7.5 g, 89%), R_f_ = 0.7; IR (KBr): *ν* (cm^−1^) 1737 (CO), 1532 (CN); ^1^H-NMR (DMSO-*d*_6_): δ 2.89 (t, *J* = 7.2 Hz, 2H, CH_2_CO), 3.61 (s, 3H, CH_3_), 4.35 (t, *J* = 7.2 Hz, 2H, NCH_2_), 6.67 (s, 1H, SCH), 7.24–7.90 (m, 10H, H_Ar_); ^13^C-NMR (DMSO-*d*_6_): δ 32.9 (CH_2_CO), 49.1 (NCH_2_), 51.8 (CH_3_), 101.9, 126.1, 127.1, 127.6, 127.7, 128.6, 130.2, 135.1, 145.1, 151.5 (C_Ar_, C_thiazole_), 169.4 (CO), 172.4 (C=N); Anal. Calcd for C_19_H_18_N_2_O_2_S: C, 67.43%; H, 5.36%; N, 8.28%; Found: C, 67.35%; H, 5.42%; N, 8.19%.

*3-[Phenyl(4-phenyl-1,3-thiazol-2-yl)amino]propanehydrazide* (**13**). A mixture of ester **12** (7.2 g, 0.02 mol), hydrazine monohydrate (2.07 g, 0.04 mol) was refluxed in 2-propanol (20 mL) for 7 h. The reaction mixture was cooled, the solvent was evaporated, and the oilseed substance was purified by chromatography on a silica gel 60 column (methanol–chloroform, 1:20), *R_f_* 0.6. Oil, (5.1 g, 71%), IR (KBr): *ν* (cm^−1^) 3283, 3060 (NH, NH_2_), 1661 (CO), 1532 (CN); ^1^H-NMR (DMSO-*d*_6_): δ 2.71 (t, *J* = 6.7 Hz, 2H, CH_2_CO), 3.53–4.06 (br s, 2H, NH_2_), 4.34 (t, *J* = 6.7 Hz, 2H, NCH_2_), 6.67 (s, 1H, SCH), 7.26–7.88 (m, 10H, H_Ar_), 8.06 (s, 1H, NH); ^13^C-NMR (DMSO-*d*_6_): δ 33.6 (CH_2_CO), 49.4 (NCH_2_), 102.3, 126.1, 126.9, 127.8, 127.9, 128.8, 130.3, 134.8, 144.7, 151.3 (C_Ar_, C_thiazole_), 170.1 (CO), 171.7 (C=N); Anal. Calcd for C_18_H_18_N_4_OS: C, 63.88%; H, 5.36%; N, 16.56%; Found: C, 63.72%; H, 5.45%; N, 16.59%.

### 3.3. General Synthesis of Hydrazones **14 b**, **c**, **i**, **j**

A mixture of hydrazide **13** (1.01 g, 3 mmol), the corresponding aldehyde (4 mmol) and 2-propanol (6 mL) was refluxed for 5 h. After cooling, the precipitated compound was filtered, washed with 2-propanol, and crystallized.

*3-[(4-Phenyl-1,3-thiazol-2-yl)(phenyl)amino]-N'-(4-nitrophenyl)methylene]propanehydrazide* (**14b**). Yellow crystals, (1.19 g, 84%), m.p. 218–219 °C (from 1,4-dioxane); IR (KBr): *ν* (cm^−1^) 3082 (NH), 1676 (CO), 1541, 1514 (CN); ^1^H-NMR (DMSO-*d*_6_): δ (*E/Z*, 40/60): 2.76, 3.18 (2 t, *J* = 7.1 Hz, 2H, CH_2_CO), 4.27–4.38 (m, 2H, NCH_2_), 7.13–8.30 (m, 15H, H_Ar_, H_tiazole_), 8.25, 8.30 (2 s, 1H, N=CH), 11.70, 11.78 (1 H, 2 s, NHN); Anal. Calcd for C_25_H_21_N_5_O_3_S: C, 63.68%; H, 4.49%; N, 14.85%; Found: C, 63.55%; H, 14.77%; N, 8.50%.

*3-[(4-Phenyl-1,3-thiazol-2-yl)(phenyl)amino]-N'-(3-nitrophenyl)methylene]propanehydrazide* (**14c**). Red-yellow crystals, (1.15 g, 81%), m.p. 147–148 °C (from 1,4-dioxane); IR (KBr): *ν* (cm^−1^) 3109 (NH), 1681 (CO), 1532, 1515 (CN); ^1^H-NMR (DMSO-*d*_6_): δ (*E/Z*, 40/60): 2.75, 3.15 (2 t, *J* = 7.1 Hz, 2H, CH_2_CO), 4.27–4.39 (m, 2H, NCH_2_), 7.10–8.36 (m, 15H, H_Ar_, H_thiazole_), 8.47, 8.50 (2 s, 1H, N=CH), 11.62, 11.73 (2 s, 1H, NHN); Anal. Calcd for C_25_H_21_N_5_O_3_S: C, 63.68%; H, 4.49%; N, 14.85%; Found: C, 66.79%; H, 4.61%; N, 14.80%.

*N* *'-[(5-Bromo-2-thienyl)methylene]-3-[(4-phenyl-1,3-thiazol-2-yl)(phenyl)amino]propanehydrazide* (**14i**). Grey crystals, (1.1 g, 72%), m.p. 144–145 °C (from 1,4-dioxane); IR (KBr): *ν* (cm^−1^) 3057 (NH), 1674 (CO), 1533, 1514 (CN); ^1^H-NMR (DMSO-*d*_6_): δ (*E/Z*, 40/60): 2.69, 3.04 (2 t, *J* = 7.1 Hz, 2H, CH_2_CO), 4.23–4.34 (m, 2H, NCH_2_), 7.09–7.93 (m, 13H, H_Ar_, H_tiazole_, H_thiophene_), 8.04, 8.28 (2 s, 1H, N=CH), 11.43, 11.49 (2 s, 1H, NNH); Anal. Calcd for C_23_H_19_BrN_4_OS_2_: C, 54.01%; H, 3.74%; N, 10.95%; Found: C, 54.15%; H, 3.82%; N, 11.03%.

*N* *'-[(5-Nitro-2-thienyl)methylene]-3-[(4-phenyl-1,3-thiazol-2-yl)(phenyl)amino]propanehydrazide* (**14j**). Orange crystals, (0.98 g, 70%), m.p. 208–209 °C (from 1,4-dioxane); IR (KBr): *ν* (cm^−1^) 3065 (NH), 1672 (CO), 1541, 1518 (CN); ^1^H-NMR (DMSO-*d*_6_): δ (*E/Z*, 40/60): 2.74, 3.08 (2 t, *J* = 7.0 Hz, 2H, CH_2_CO), 4.25–4.37 (m, 2H, NCH_2_), 7.11–8.11 (m, 13H, H_Ar_, H_thiazole_, H_thiophene_), 8.12, 8.38 (2 s, 1H, N=CH), 11.79, 11.83 (2 s, 1H, NNH); Anal. Calcd for C_23_H_19_N_5_O_3_S_2_: C, 57.85%; H, 4.01%; N, 14.66%; Found: C, 57.77%; H, 4.14%; N, 14.71%.

### 3.4. Antimicrobial Activity

The following bacteria strains were used: gram-negative rods of *Escherichia coli* (ATCC 8739), *Pseudomonas aeruginosa* (NCTC 6750), gram-positive cocci of *Staphylococcus aureus* (ATCC 9144) and gram-positive spore forming rods of *Bacillus cereus* (ATCC 11778). Tryptic soy agar (TSA) and tryptic soy broth (TSB) were used for bacteria cultivation and antibacterial activity tests.

Antibacterial activity of the compounds was determined by testing their different concentrations against *B. cereus*, *S. aureus, P. aeruginosa* and *E. coli* bacteria by the broth-dilution and spread plate methods [39,40]. A range of concentrations, 1000, 500, 350, 250, and 125 µg/mL were prepared for each sample. They were streaked out on TSA plates and incubated at 37 °C for 24 h. A representative colony was placed in 5 mL of TBS and incubated at 37 °C for 24 h.

*B. cereus, S. aureus, E. coli*, and *P. aeruginosa* cultures containing 10^8^ CFU/mL (colony-forming units corresponding to McFarland’s 0.5) were diluted with TSB and used for the antibacterial test. The test organisms (100 µL) were added to each tube and incubated at 37 °C for 24 h. At the end of this period, a small amount of the diluted mixture (different materials) from each tube was pulled out and spread on TSA. The plates were incubated at 37 °C for 48 h. The growth of bacterial cells was observed on agar plates. The lowest concentration of the bacterial material at which no growth was observed was considered as the minimum bactericidal concentration (MBC) value [41]. The antibacterial compound oxytetracycline inoculated with each test bacterium in the tubes and plates was used as a control. The growth of the test bacteria was observed in all plates as positive controls.

### 3.5. Field Trials and Rapeseed Biochemical Ontent

The influence of N-substituted β-alanine derivatives on the growth of rapeseed (*Brassica napus* L.) and St. John wort (*Hypericum perforatum* L.) *in vitro* was explored. According to the results, 3-[(4-oxo-4,5-dihydro-1,3-thiazol-2-yl)(phenyl)amino]propanoic acid (**2**) was selected, because it had the most remarkable positive effect on the growth of rapeseed and St. John wort *in vitro* [34]. Following the data obtained during the *in vitro* experiments, compound **2** was selected for the field trials.

Experiments with 3-[(4-oxo-4,5-dihydro-1,3-thiazol-2-yl)(phenyl)amino]propanoic acid on spring rapeseed (*Brassica napus* L.) of the SW Landmark variety were carried out in field trials in 2012 at the Lithuanian Research Centre for Agriculture and Forestry branch Rumokai experimental station. Spring rapeseed was sprayed with the study compound solutions (concentrations 25–150 mg/L) before flowering. For each field, 1 L of the solution was used. The soil of the experimental field was carbonaceous shallow luvisols (Calc or epihypogleyic luvisol IDg8-k (*LVg-w-cc*) with the following charateristics: pH_KCl_ 6.7, humus 1.82%, N_total_ 0.12%, 308 mg kg^−1^ of mobile P_2_O_5_, 296 mg kg^−1^ of mobile K_2_O. Soil samples were collected on 25.04.2012. Barley was the preceding crop. The size of the initial and accounted fields was 2.7 × 11 m and 2.2 × 10 m, respectively. Experiments with rapeseed were performed in April–August. The average air temperature ranged from 7.9 to 19.3 °C, the rainfall ranged from 44.4 to 118.4 mm per month, and during the experiment it was 361.1 mm. The rainfall was by 11.1 mm higher than the average multi-annual precipitation. Biometric measurements were carried out after harvesting. The moisture of rape seeds was 8.5%. The oil content in rape seeds was measured by extraction with hexane for 3 hours in a Soxhlet apparatus (Behr Labor-Technik, Dusseldorf, Germany). Fatty acid composition was analysed with a HRGC 5300 Mega Series gas chromatograph Carlo Erba Strumentazione (Milano, Italy). The protein content in rape seeds was measured by the Bradford method.

## 4. Conclusions

The *N*-phenyl-*N*-thiocarbamoyl-β-alanine was used for the first time for the synthesis of biologically active 2-aminothiazole derivatives. By a new method of synthesizing the target structures we have established a convenient way to produce a number of compounds, which were used for biological testing. Some of the synthesized compounds exhibited promising antimicrobial activity, and 3-[(4-oxo-4,5-dihydro-1,3-thiazol-2-yl)(phenyl)amino]propanoic acid (**2**) was found to promote the growth, to increase seed yield and oil content in rapeseed.
